# Inflammatory and non-inflammatory monocytes as novel prognostic biomarkers of survival in SOD1G93A mouse model of Amyotrophic Lateral Sclerosis

**DOI:** 10.1371/journal.pone.0184626

**Published:** 2017-09-08

**Authors:** Samanta Gasco, Pilar Zaragoza, Alberto García-Redondo, Ana C. Calvo, Rosario Osta

**Affiliations:** 1 LAGENBIO, Veterinary Faculty of Zaragoza, Instituto Agroalimentario de Aragón (I2A), CITA, Health Research Institute of Aragon (IIS). University of Zaragoza, Zaragoza, Spain; 2 Biochemistry Department, CIBERER U-723. Health Research Institute, October 12th Hospital, Madrid, Spain; University of Rome La Sapienza, ITALY

## Abstract

Amyotrophic Lateral Sclerosis (ALS) has lately become a suitable scenario to study the interplay between the hematopoietic system and disease progression. Recent studies in *C9orf72* null mice have demonstrated that *C9orf72* is necessary for the normal function of myeloid cells. In this study, we aimed to analyze in depth the connection between the hematopoietic system and secondary lymphoid (spleen) and non-lymphoid (liver and skeletal muscle) organs and tissues along the disease progression in the transgenic SOD1G93A mice. Our findings suggested that the inflammatory response due to the neurodegeneration in this animal model affected all three organs and tissues, especially the liver and the skeletal muscle. However, the liver was able to compensate this inflammatory response by means of the action of non-inflammatory monocytes, while in the skeletal muscle inflammatory monocytes prompted a further inflammation process until the terminal state of the animals. Interestingly, in blood, a positive correlation was found between non-inflammatory monocytes and survival of the transgenic SOD1G93A mice, while the contrary (a negative correlation) was found in the case of inflammatory monocytes, supporting their potential role as biomarkers of disease progression and survival in this animal model. These findings could prompt future translational studies in ALS patients, promoting the identification of new reliable biomarkers of disease progression.

## Introduction

Amyotrophic Lateral Sclerosis (ALS) is a late-onset fatal neurodegenerative disease characterized by a progressive loss of upper and lower motoneurons, resulting in weakness, muscle atrophy, spasticity, paralysis and, ultimately, death of the patients in one to five years on average from the onset of the disease [1]. ALS has two forms, sporadic ALS (SALS, involving 90–95% of the cases) and familial ALS (FALS, involving 5–10% of the cases). Regarding FALS, several gene mutations have been found to cause it. Classicaly, mutations in the *SOD1* gene were the most commonly found (around 20% of FALS cases), but the recent discovery of mutations in the *C9orf72* gene put the latter as the most common mutation causing FALS, being found in 20–50% of the cases. Mutations in other genes have also been linked to FALS to a lesser extent, such as *TARDBP*, *FUS*, *ANG*, *OPTN* and *SETX* [2]. On the other hand, the etiology of SALS remains unclear, with no familial history being found in this ALS form. A few of SALS cases have a connection with the aforementioned genes but the vast majority of cases have an unclear underlying cause [2–4]. ALS is considered a multi-factorial neurodegenerative disease where the interplay of many pathological mechanisms initiate motoneuron neurodegeneration. Some of the mechanisms related to the pathogenesis underlying ALS are oxidative stress, glutamate-mediated excitotoxicity, mitochondria abnormalities and impaired axonal transport [5–8], though it is not clear if these mechanisms initiate the disease or are secondary processes following the initial insult [9]. Following the discovery of the ALS causing genes, several mice models for ALS were generated. The most commonly used mice model is the transgenic mouse overexpressing mutant (G93A) SOD1. However, other mice models exist, such as SOD1 trangenic mice containing other mutations (G37R, G85R, D90A or G86R) and TDP-43, FUS-TLS, ALS2, VAPB and dynactin mutant transgenic mice, among others. These mice models mimic some but not all the aspects of human ALS. They have proved to be useful for improving knowledge on the disease pathological mechanisms and as screening tools for testing potential therapies but, unfortunately, no totally successful biomarker or therapy derived from their study has arisen [4, 10]. To date, there is no curative treatment for ALS and the diagnosis usually comes late, which difficults the application of palliative measures, as well as the prognosis and the effective inclusion of the patients in clinical trials [2].

The hematopoietic system, with their central cells, the Hematopoietic Stem and Progenitor Cells (HSPCs) and, more specifically, the immune system, does not stay away from the pathological events occurring in ALS. The dual role of both the innate and the adaptive immune system has been largely studied in ALS. It is accepted that the immune response starts as a debris-cleaning and reparative mechanism at the beginning of the disease, and exacerbates its action towards the terminal phase, when an inflammatory immune phenotype prevails, thus causing more harm than good in the damaged tissues [11, 12].

Monocytes have an important role in the immune response dualism occurring in ALS, as they are part of the innate immune system, and are one of the first cell types to sense tissue damage and to be rapidly recruited from peripheral blood to the affected tissues at the beginning of the tissue insult [13]. Their function dualism relies on the possibility of generating two different subsets with very different functions. In steady state conditions, classical/inflammatory Ly6C^hi^ (Gr-1+) monocytes (from now on called inflammatory monocytes) patrol the extravascular tissues, picking up antigens and entering the lymph nodes to present the antigens to lymphocytes, and they usually remain undifferentiated. In pathological conditions, however, they are rapidly recruited into the damaged and inflamed tissues in a CCR2-dependent manner, as the majority of Ly6C^hi^ inflammatory monocytes express the CCR2 receptor [14, 15]. On the other hand, the non-classical/non-inflammatory Ly6C^lo^ (GR-1-) monocytes (from now on called non-inflammatory monocytes) express little or no CCR2 receptors and high levels of CX3CR1 receptor. It is usually accepted that the majority of the non-inflammatory monocytes derive from Ly6C^hi^ monocytes, as the latter are capable of generating this non-inflammatory subset in steady state conditions in peripheral blood [15, 16]. The main function of this monocyte subset is to patrol the luminal side of the endothelium searching for and clearing damaged endothelial cells and debris to maintain vasculature integrity. In pathological conditions, though, non-inflammatory monocytes are capable of entering the inflamed tissues and they likely differentiate to M2 type (non-inflammatory) macrophages, which possess the ability to secrete anti-inflammatory cytokines and also to repair the damaged tissue [16]. Inflammatory monocytes, however, are more likely to differentiate to M1-type inflammatory macrophages, which secrete pro-inflammatory cytokines able to combat pathogenic microorganisms, but ultimately harmful in neurodegenerative diseases like ALS [11]. In ALS, non-inflammatory monocyte/macrophages act from the beginning of the disease in an effort to remove the debris and damage derived from motoneuron degeneration and death, as well as promoting regeneration. These cells are observed in big numbers in the CNS at the beginning of the pathological processes involved in ALS [11]. MCP-1 (also known as CCL2) ligand was observed to increase in spinal cord of ALS mice models along disease progression [17], thus meaning that monocytes/macrophages are highly recruited at this stage of the disease. CCL2 was also found to be increased in plasma from human patients [18]. Also, in the peripheral nervous system, degeneration of peripheral motor axons was found at the early stages of ALS in patients and mouse models and is preceeded by the recruitment and activation of macrophages [19]. At the symptomatic and terminal stages of ALS, however, the monocyte/macrophage population phenotype changes, as inflammatory monocytes/macrophages are predominantly recruited to the CNS. At this phase, monocytes and their progeny, together with other immune cells, create a pro-inflammatory environment further damaging motoneurons, given the impossibility to stop neurodegeneration at the first stages of the disease [11]. In fact, blocking Ly6C+ monocytes in the SOD1G93A mice model resulted in delayed disease symptoms and extended lifespan [20], thus confirming the exacerbation role of inflammatory monocytes/macrophages at these stages. However, little is known about the role and potential presence of monocytes in other tissues during the course of ALS, including the skeletal mucle, which one of the most affected tissues in the disease.

As aforementioned, the lack of reliable biomarkers in ALS prevents from an early diagnosis and prognosis of the disease. For this reason, and given the relevance of the hematopoietic and immune system in ALS pathological events, the aim of the present work was to study the role of Hematopoietic Stem and Progenitor Cells (HSPCs) as well as inflammatory and non-inflammatory monocytes along disease progression in blood, spleen, liver and skeletal muscle of the SOD1G93A mice model to provide, not only a better understanding of the disease mechanisms, but also the potential identification of novel biomarkers which could be serially and non-invasively monitored for an easier translation to ALS patients. To reach this objective, HSPC and monocyte populations were studied, characterized and quantified by means of flow cytometry, RT-PCR and Western Blot techniques.

## Materials and methods

### Animals

Wild-type (WT) mice on a B6SJL genetic background and SOD1G93A mutant mice on a mixed B6SJL background used for the experimental procedures were provided by The Jackson Laboratory (Bar Harbor, Maine, Sacramento, California). Three groups were formed; the first one was composed of 12 WT and 12 SOD1G93A age and sex-matched mice per time point and were used to perform the study of HSPCs in spleen and monocyte populations in spleen, liver and skeletal muscle. The second group was composed of 24 mice, 12 WT and 12 SOD1G93A age and sex-matched mice, and were used for the serial study of monocyte populations in blood. Finally, the third group was also composed by 12 WT and 12 SOD1G93A age and sex-matched mice and was used for the serial study of the release efficiency (Input Rate, IR) of HSPCs to the blood along disease progression. All the experimental procedures were approved by the Ethic Committee for Animal Experiments of the University of Zaragoza. Animal care and experimentation were performed accordingly with the Spanish Policy for Animal Protection RD53/2013, which meets the European Union Directive 2010/63/UE on the protection of animals used for experimental and other scientific purposes. Animals were housed in isolated cages with individual ventilation (Techniplast) in the Unidad Mixta de Investigación of the University of Zaragoza under a 12h light/night cycle. Ambiental conditions included a stable temperature of 21–23°C and a relative humidity of 55%. Food and water was administered *ad libitum*.

### Peripheral blood obtaining for flow cytometry

12 SOD1G93A mice (6 males and 6 females) and 12 WT mice were used for the flow cytometry analysis. Blood extractions were performed serially at 50 and 75 postnatal (P) days and at sacrifice. The sacrifice point for every mouse was established by performing a test where the mouse was placed in supine position; if the mouse failed to right itself within 30 seconds, it was considered to be terminal and in need of sacrifice (disease endpoint) [21]. Mice tails were exposed to an infrared light to dilate the tail veins. Blood was then collected by puncturing one of the tail veins using a 25G needle and collecting the blood drops with Microvette® CB 300 (Sarstedt; Nümbrecht, Germany) capillary tubes containing EDTA. The average amount of blood obtained was 40 μL, which proved to be enough for the flow cytometry procedure [22].

### Spleen unicellular suspension preparation for flow cytometry

12 SOD1G93A mice and 12 WT mice (6 females and 6 males for each group) were sacrificed with CO2 anesthesia and spleens were carefully extracted and washed briefly with PBS at each of the selected time points (P50, P75 and P120). Immediately after, spleens were mashed and pressed through a 70 μM strainer into a PBS containing tube and centrifuged at 400 x g for 10 minutes. Erythrocytes were lysed with an ammonium chloride lysing reagent and samples were centrifuged again at 400 x g for 5 minutes. The supernatant was then removed and the pellet was resuspended in PBS for further flow cytometry analysis.

### Flow cytometry

Blood and spleen single cell samples were washed with Flow Cytometry Staining Buffer (eBioscience; San Diego, California, USA) and surface-stained. Blood monocytes were stained using the following antibodies: FITC-conjugated anti-Integrin αM/CD11b antibody, PE-conjugated anti-CCR2 antibody, APC-conjugated anti-CX3CR1 antibody and PerCP-conjugated anti-GR-1/Ly6G (R&D Systems, Minneapolis, USA). For the identification of spleen HSPCs the following antibodies were used: Mouse Hematopoietic Lineage eFluor 450 Cocktail containing CD3 (17A2), CD45R (B220), CD11b (m1/70) TER-119 (TER-119) and Ly-G6 (Gr-1) (RB6-(C5)) antibodies; PE-conjugated anti-Ly-6A/E (Sca-1) antibody and APC-conjugated anti-CD117 (c-Kit) antibody (eBioscience; San Diego, California, USA); and Pe-Cy7-conjugated anti-CD127 (IL7RA) antibody (BD Biosciences; Franklin Lakes, New Jersey, USA). Erythrocytes were lysed and samples were washed and resuspended in PBS for analysis with Gallios Flow Cytometer (Beckman Coulter; Brea, California, USA). Total blood monocytes were identified as SSC^mid^FSC^hi^CD11b+. Although CD115 appears to be a more specific marker to specifically identify monocytes, processing time is a limiting factor, as Breslin et al. demonstrated that to obtain reliable results, samples should be analyzed in less than two hours, and if this is not possible, CD11b is a good alternative [22]. In our case, the extraction, processing and analysis of the samples took more than two hours due to the need to use an external flow cytometry service, so CD115 was not the best option for us and CD11b was selected instead. Classical/inflammatory monocytes were characterized as CD11b+Gr-1+ and further purified by identification of CCR2+CX3CR1- monocytes. Non-classical/non-inflammatory monocytes were defined as CD11b+Gr-1- and further analyzed as CCR2-CX3CR1+ [23–27]. HSCs in the spleen were identified as Lin-c-Kit+Sca-1+, CLPs as Lin-c-Kit^low^Sca-1^low^CD127+, and CMPs as Lin-c-Kit+Sca-1-CD127- [28, 29]. Data was obtained and analyzed using Kaluza Flow Analysis Software (Beckman Coulter; Brea, California, USA). Spectral overlap was corrected for every antibody using compensation controls. Negative controls were used to correct non-specific antibody fluorescence. HSPCs and monocytes were studied and quantified by generating the necessary density plots, dot plots and histograms, and the data obtained was exported to an Excel sheet (Microsoft; Redmond, Washington, USA) for a further assessment and final statistical analysis.

### mRNA extraction and assessment of gene expression by RT-PCR

12 SOD1G93A mice and 12 age and sex-matched WT mice (sex balanced for the two genotypes) were used for this experimental procedure. The mice were sacrificed with CO2 anesthesia and the *quadriceps femoris* muscles, the liver and the spleen from every animal were extracted and washed briefly with PBS at P50, P75 and P120. Tissue was frozen with liquid nitrogen and crushed with Cell Crusher pulverizer (Stratech Scientific Ltd., Suffolk, UK). Part of the powder was frozen at -80°C for later use for Western Blot. For RNA extraction, tissue powder was treated following the Trizol-Chloroform method. RNA was subsequently treated with Turbo DNA-free kit (Ambion®, Life Technologies; Waltham, Massachussets, USA) to eliminate genomic DNA. Once purified, reverse transcription was performed according to SuperScript First-Strand Synthesis System kit protocol (Invitrogen™, Life Technologies; Waltham, Massachussets, USA). Gene expression was analyzed with StepOne™ Real-Time PCR System (Life Technologies; Waltham, Massachussets, USA). *Ly6C* (Ly6c, Mm03009946_m1), *Ccr2* (CCR2, Mm01216173_m1) and *Cx3cr1* (CX3CR1, Mm004138354_m1) probes were supplied by Applied Biosystems® (Life Technologies; Waltham, Massachussets, USA). *Gapdh* (GAPDH, Mm03302249_g1, Applied Biosystems®, Life Technologies; Waltham, Massachussets, USA) and *β-actin* (ACTB, 4352933, Thermo Fisher; Waltham, Massachussets, USA) were used as *housekeeping* genes for normalization of the data obtained. The relative gene expression obtained from this normalization was assessed through the use of the 2^-ΔΔCT^ method [30].

### Protein extraction and Western blot analysis

The *quadriceps femoris* muscle, liver and spleen powder fraction preserved for protein analysis (from the same mice sacrificed for mRNA obtaining) was resuspended in RIPA lysis buffer with protease inhibitors (Roche, Basel, Switzerland), homogenated and centrifuged. Supernatants were collected and total protein was quantified using the BCA method (Sigma-Aldrich, San Luis, Misuri, United States). After quantification, 25 μg of protein were loaded to each lane of a 8% SDS-page gel. After proper protein resolving, proteins were transferred to a PVDF membrane (Amersham™, GE Healthcare Life Sciences, Little Chalfont, United Kingdom) and subsequently blocked with a Tris-buffered saline solution containing 5% skimmed milk and 0.1% Tween as supplement for 1 hour at room temperature. Membranes were then incubated overnight at 4°C with the selected primary antibodies: anti-CKR-2 antibody (sc-30032, Santa Cruz Biotechnology, Santa Cruz, California, USA), anti-CX3CR1 antibody (sc-30030, Santa Cruz Biotechnology, Santa Cruz, California, USA) and anti-Ly-6C (sc-23080, Santa Cruz Biotechnology, Santa Cruz, California, USA). GAPDH (sc-25778, Santa Cruz Biotechnology, Santa Cruz, California, USA) was selected as normalization protein. Incubation with HRP-conjugated secondary antibodies was performed the next day for 1 hour at room temperature, and bands were visualized using ECL reagents (GE Healthcare Life Science). Inmunoblots were scanned and a densitometry study was performed to measure band intensity with AlphaEase FC software (Bonsai).

### Measurement of HSPCs release efficiency by calculation of Input Rate (IR) parameter

The efficiency of the bone marrow to release HSCs, CLPs and CMPs to the blood stream was calculated by creating a new parameter we named Input Rate (IR). This ratio was calculated with the following expression:

$$\frac{N_{x}-N_{x-1}}{N_{T}(t_{x}-t_{x-1})}$$

Where *N* is the cell count of the population studied (HSC, CLPs or CMP), *N*_*T*_ is the total cell number *x* is the extraction point and *t* is time (in days). In this experiment, both WT and SOD1G93A mice were monitored serially starting at a very early asymptomatic stage (P30) to assure an accurate measurement of HSPCs along disease progression. Each cell population was measured by flow cytometry before calculation of the IR. This way, we obtained four IRs: IR1, IR2, IR3 and IR4. So, IR1 was calculated by subtracting the cell percentage at P30 to the cell percentage at P50 and then dividing the value obtained by the days elapsed between both blood extractions; and the same applied to IR2, IR3 and IR4.

### Statistical analysis

Data normality and homogeneity of variance were assessed with Kolmogórov-Smirnov and Levene tests, respectively. Cell percentage means for the study of HSPCs and monocytes in the different tissues were compared using two-way independent ANOVA statistical test, which allowed to assess the main effects and interaction of genotype and the different time points selected on cell percentages, mRNA expression and protein contents. However, for IRs and monocyte means, a two-way mixed ANOVA was performed, as the mice were the same along time points. Further interaction effects between genotype and time points on the dependent variable were assessed with pairwise comparisons also provided by the two-way ANOVAs calculated. Correlations between total, inflammatory and non-inflammatory monocyte slopes and SOD1G93A mice survival were calculated using Pearson’s *r*. The individual progression and survival of each mouse was taken into account, as individual slopes were calculated for each mouse by fitting a linear regression model and then incorporated in the Pearson’s *r* coefficients calculation. Also, this way, the statistical study was not biased by animals presenting a shorter or longer lifespan than the animals with an average lifespan. The statistical analysis was performed using SPSS Statistics version 19.0 software (IBM; Armonk, New York, USA). The data are presented as means, and error bars represent the standard error of the mean (s.e.m.) for flow cytometry and Western Blot results, while error bars for RT-PCR results were calculated as described in the 2^-ΔΔCT^ method [30]. Statistical significance was established at *p*-values under 0.05 (*), high statistical significance at *p*-values under 0.01 (**) and very high statistical significance at *p*-values under 0.001 (***).

## Results

### HSPCs commitment and differentiation to the myeloid lineage at the terminal stage in the spleen

**Fig 1:**
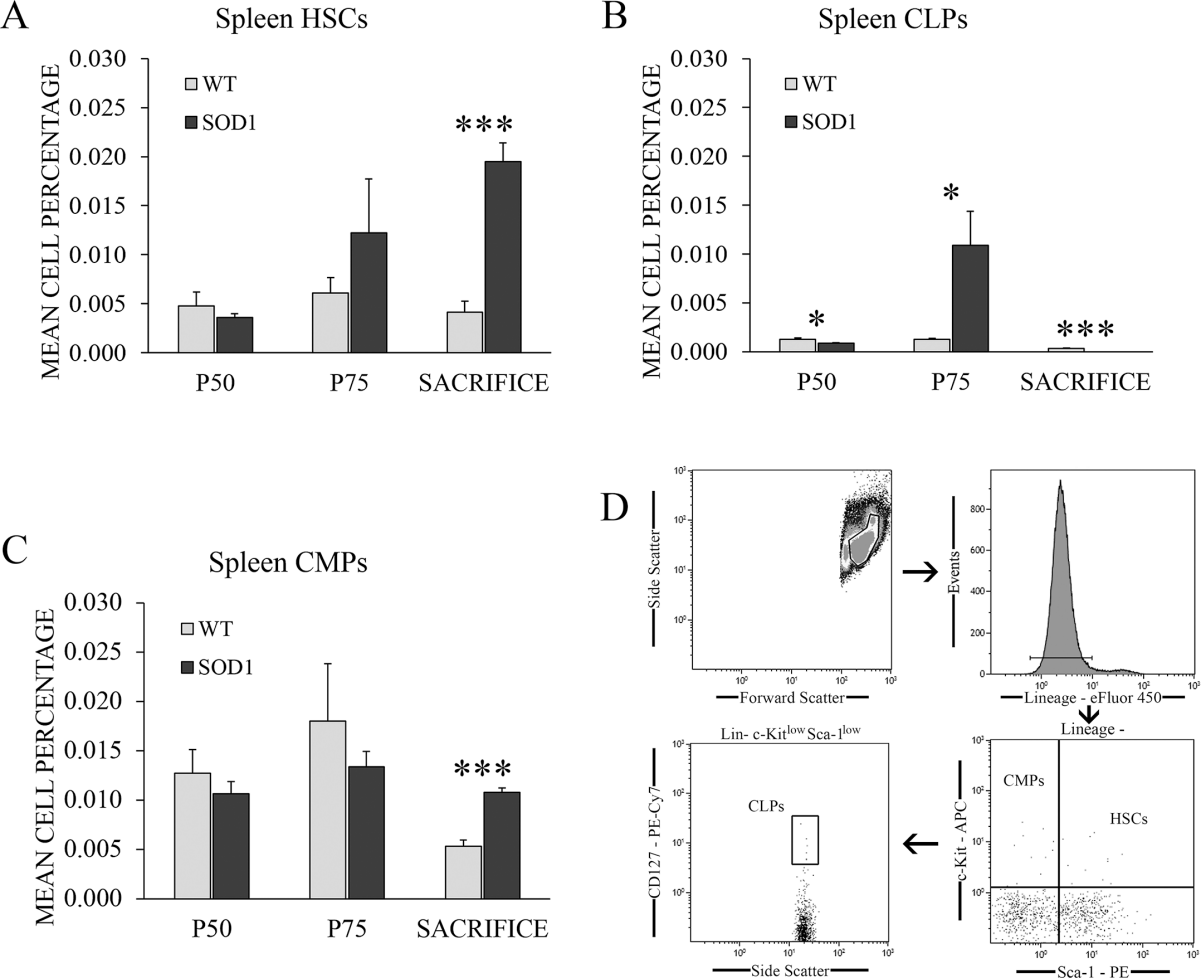
HSPCs percentages recorded by flow cytometry in the spleen of WT and transgenic SOD1G93A mice. **(A)** HSC, **(B)** CLP and **(C)** CMP percentages were measured by flow cytometry in the spleen of transgenic SOD1G93A mice (black bars) at the main stages of the disease: asymptomatic stage (P50), symptomatic stage (P75) and terminal stage (sacrifice). WT litter mates (light grey bars) were used as controls. The gating strategy of the flow cytometry analysis is shown in **(D)**. *y*-axis represents the mean cell percentage for each cell type, and *x*-axis represents the time points selected. Data are represented as means and error bars represent the standard error of the mean (s.e.m.). Statistical significance was established at *p*-values under 0.05 (*), high statistical significance at *p*-values under 0.01 (**) and very high statistical significance at *p*-values under 0.001 (***). n = 12 animals per genotype and time point.

Cell percentages of HSCs, CLPs and CMPs were analyzed by flow cytometry in the spleen of transgenic SOD1G93A mice at the asymptomatic (P50), symptomatic (P75) and terminal stage of the disease (sacrifice) (Fig 1), following the analysis flow shown in Fig 1D. Results from the two-way independent ANOVA showed a significant effect of the interaction between genotype and time point variables for all three cell types studied (F(2,66) = 8.127, *p* < 0.01, η^2^ = 0.351 for HSCs, F(2,66) = 5.51, *p* < 0.05, η^2^ = 0.306 for CLPs and F(2,66) = 5.73, *p* < 0.05, η^2^ = 0.305 for CMPs). Further pairwise comparison analysis obtained from the two-way independent ANOVA calculation found a significant increase of HSCs and CMPs percentages in transgenic SOD1G93A mice with respect to control mice at sacrifice (*p* < 0.001) (Fig 1A and 1C), and at P75 for CLPs (*p* < 0.05) (Fig 1B). However, a significant downregulation of CLPs percentage was detected at P75 (*p* < 0.05) and sacrifice in transgenic SOD1G93A mice (Fig 1B). In particular, CLPs were completely absent at P120 in all the SOD1G93A mice (*p* < 0.001). The upregulated percentages of HSCs and CMPs could suggest a myeloid differentiation of HSCs in this tissue in the late stage of the disease, in which a complete absence of CLPs was simultaneously observed. Considering the relevant role of the spleen in the hematopoietic system, our next step was to analyze if the CMPs upregulation could also indicate a deregulation of the monocyte population in this tissue as a consequence of disease progression.

### Absence of inflammatory response in the spleen of transgenic SOD1G93A mice

**Fig 2:**
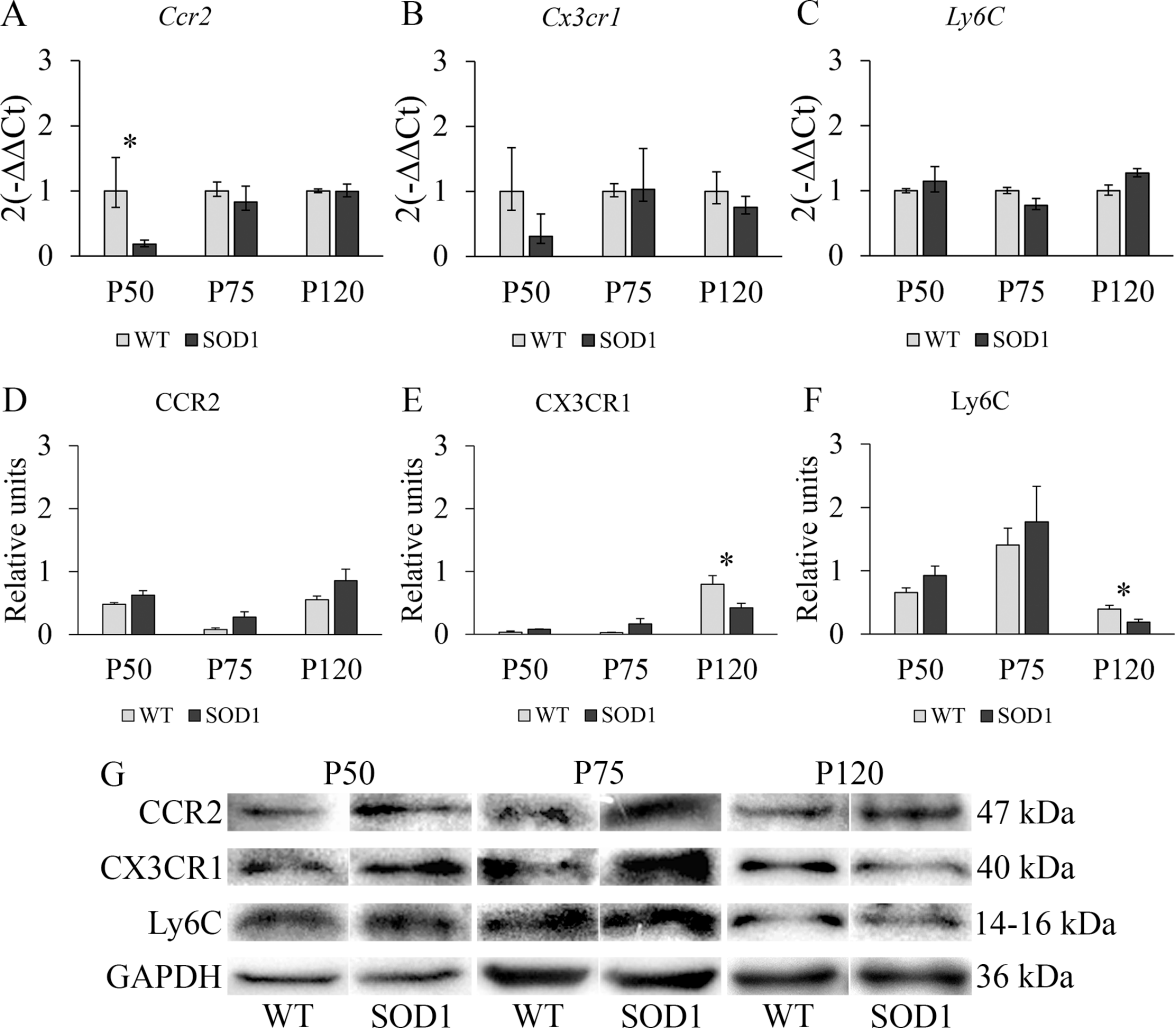
RT-PCR and Western blot study of monocytic markers CCR2, CX3CR1 and Ly6C in the spleen of WT and transgenic SOD1G93A mice. **(A, B, C)** RT-PCR was used to measure mRNA expression levels of *Ccr2*, expressed by inflammatory monocytes; *Cx3cr1*, a marker of non-inflammatory monocytes; and *Ly6C*, highly expressed in inflammatory monocytes. **(D, E, F)** Western blot analysis of CCR2, CX3CR1 and Ly6C protein expression levels. *y*-axis and *x*-axis represent the gene expression fold change and time points selected for RT-PCR analysis, and protein fold change and time point selected for Western Blot analysis, respectively. Transgenic SOD1G93A mice are represented with black bars and WT litter mates used as controls, with light grey bars. Error bars represent the standard error of the mean (s.e.m.) for the Western blot analysis and error bars for the RT-PCR analysis were calculated using the method developed by Livak et al. [30]. Statistical significance was established at *p*-values under 0.05 (*). n = 12 animals per genotype and time point. **(G)** Representative protein bands for every protein studied by Western blot in both WT and SOD1G93A mice. GAPDH was used as housekeeping protein for protein normalization.

To further investigate the CMP increase observed at the terminal stage (P120) in the spleen of transgenic SOD1G93A mice, we analyzed in this tissue the gene and protein levels of CCR2, CX3CR1 and Ly6C at P50, P75 and P120 (Fig 2). These molecular markers are related to the characterization of the two main subpopulations of monocytes in mice, inflammatory monocytes and non-inflammatory monocytes. In this sense, we wanted to study if the increased population of CMPs observed by flow cytometry could also be translated into an increase of inflammatory monocytes in this animal model. A significant interaction effect between genotype and time point variables was found for *Ccr2* (F(2,66) = 4.78, *p* < 0.05, η^2^ = 0.274) gene and for CX3CR1 (F(2,66) = 5.61, *p* < 0.05, η^2^ = 0.360) and Ly6C (F(2,66) = 4.83, *p* < 0.05, η^2^ = 0.382) proteins. Interestingly, downregulated mRNA levels of *Ccr2* were observed in the transgenic SOD1G93A mice at P50 (*p* < 0.05) (Fig 2A) although CCR2 protein levels remained unchanged in all the stages of the disease (Fig 2D), suggesting that the population of inflammatory monocytes was not altered in this tissue. In addition, no differences were detected in *Cx3cr1* mRNA levels (Fig 2B), while CX3CR1 protein levels were found to be significantly downregulated only at P120 (*p* < 0.05) (Fig 2E). Similarly, significantly downregulated Ly6C protein levels were also observed at P120 (*p* < 0.05) (Fig 2F). CX3CR1 can ensure the survival of infiltrating monocytes and influence their differentiation toward tissue-resident macrophages, thus enhancing inflammation and recruitment of Ly6C^hi^ monocytes if CX3CR1 is absent [31]. In light of these results, we next studied these molecular markers in the liver, which plays a relevant role in the crosstalk between hematopoietic cells and liver parenchymal cells at steady state and during injury.

### Restorative response in the liver of transgenic SOD1G93A mice

**Fig 3:**
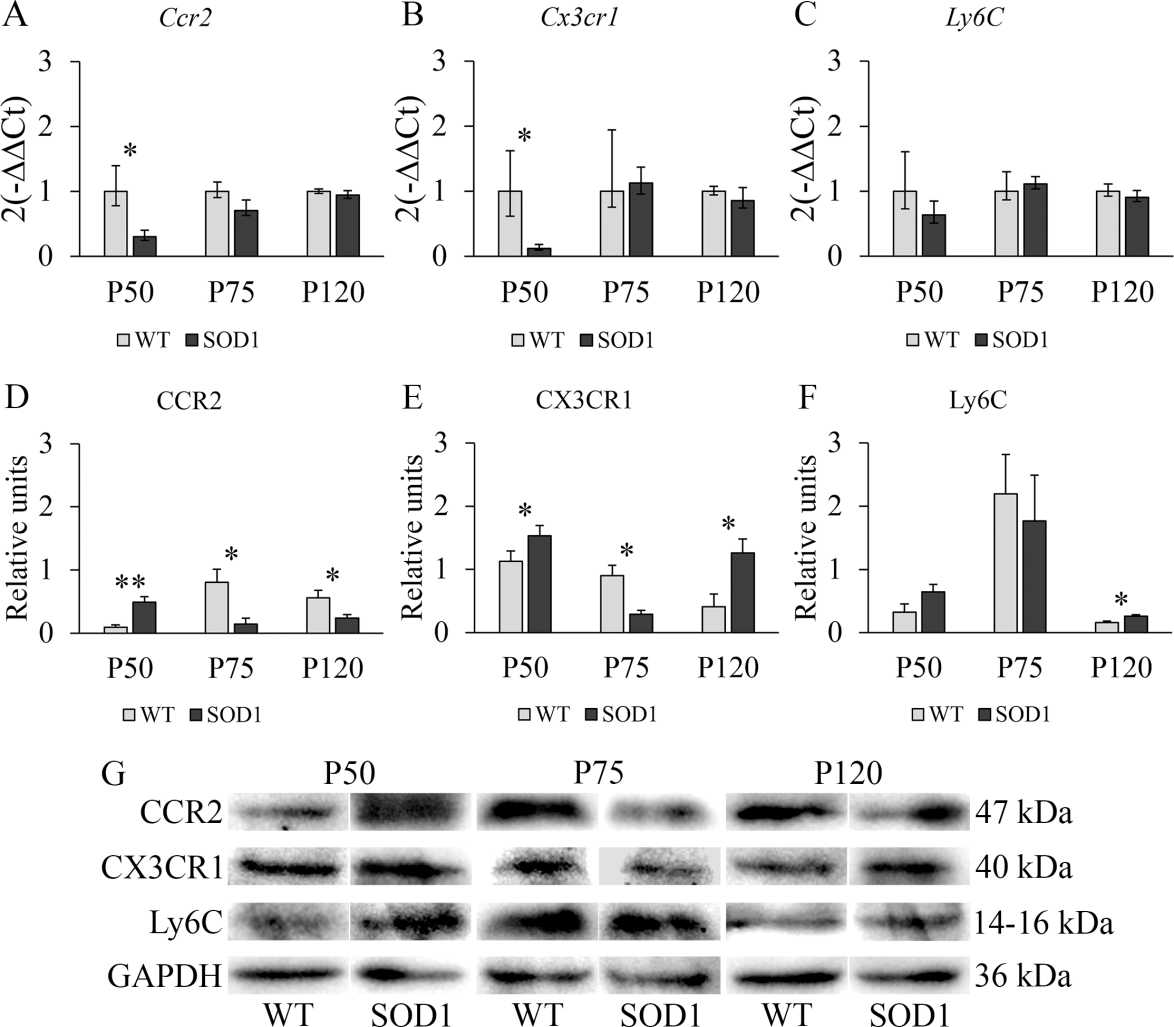
RT-PCR and Western blot study of monocytic markers CCR2, CX3CR1 and Ly6C in the liver of WT and transgenic SOD1G93A mice. **(A, B, C)** RT-PCR was used to measure mRNA expression levels of *Ccr2*, expressed in inflammatory monocytes; *Cx3cr1*, a marker of non-inflammatory monocytes; and *Ly6C*, highly expressed in inflammatory monocytes. **(D, E, F)** Western blot analysis of CCR2, CX3CR1 and Ly6C protein expression levels. *y*-axis and *x*-axis represent the gene expression fold change and time points selected for RT-PCR analysis, and protein fold change and time point selected for Western Blot analysis, respectively. Transgenic SOD1G93A mice are represented with black bars and WT litter mates used as controls, with light grey bars. Error bars represent the standard error of the mean (s.e.m.) for the Western blot analysis and error bars for the RT-PCR analysis were calculated using the method developed by Livak et al. [30]. Statistical significance was established at *p*-values under 0.05 (*) and high statistical significance at *p*-values under 0.01 (**). n = 12 animals per genotype and time point. **(G)** Representative protein bands for every protein studied by Western blot in both WT and SOD1G93A mice. GAPDH was used as housekeeping protein for protein normalization.

The results from the two-way independent ANOVA indicated that a significant interaction effect between genotype and time point variables existed for *Ccr2* (F(2,66) = 5.63, *p* < 0.05, η^2^ = 0.349) and *Cx3cr1* (F(2,66) = 5.22, *p* < 0.05, η^2^ = 0.332) genes and for CCR2 (F(2,66) = 9.07, *p* < 0.01, η^2^ = 0.476), CX3CR1 (F(2,66) = 7.85, *p* < 0.01, η^2^ = 0.440) and Ly6C (F(2,66) = 5.12, *p* < 0.05, η^2^ = 0.310) proteins. *Ccr2* and *Cx3cr1* mRNA levels in the liver decreased significantly at P50 (*p* < 0.05) (Fig 3A and 3B), while no differences were observed in *Ly6C* mRNA levels in the transgenic SOD1G93A mice (Fig 3C). Interestingly, a significant upregulation was observed in CCR2 and CX3CR1 protein levels at P50 (*p* < 0.01 for CCR2 and *p* < 0.05 for CX3CR1) (Fig 3D and 3E), suggesting the presence of both inflammatory and non-inflammatory monocytes. In contrast, at P75, the downregulated CCR2 and CX3CR1 protein levels (*p* < 0.05) (Fig 3D and 3E) could indicate a reduction of inflammatory monocytes potentially be due to their exit to the blood or to their differentiation to inflammatory macrophages, which is in accordance with the progression of the disease. However, and contrary to the response observed in the spleen, at P120, the downregulated CCR2 protein levels (*p* < 0.05) (Fig 3D) suggested that the inflammatory monocytes were possibly differentiating into inflammatory macrophages, due to decreased levels of CCR2 [32] but slightly increased Ly6c levels found (*p* < 0.05) (Fig 3F) in transgenic SOD1G93A mice. However, this fact should be further addressed with specific techniques aimed at confirming this differentiation. In addition, the increase in the levels of CX3CR1 could indicate that non-inflammatory monocytes were also entering the liver. Non-inflammatory macrophages in the liver can be originated after liver injury from non-inflammatory monocytes, and they possess a reparative function. In this sense, the liver of the transgenic SOD1G93A mice seemed to operate in a regulatory loop along disease progression. In connection with the regenerative capacity of different tissues, the skeletal muscle is mostly affected by the disease progression in this animal model. At this step, we wondered if a similar response could be found in this tissue.

### Skeletal muscle tissue in transgenic SOD1G93A mice was not able to compensate for the inflammatory response

**Fig 4:**
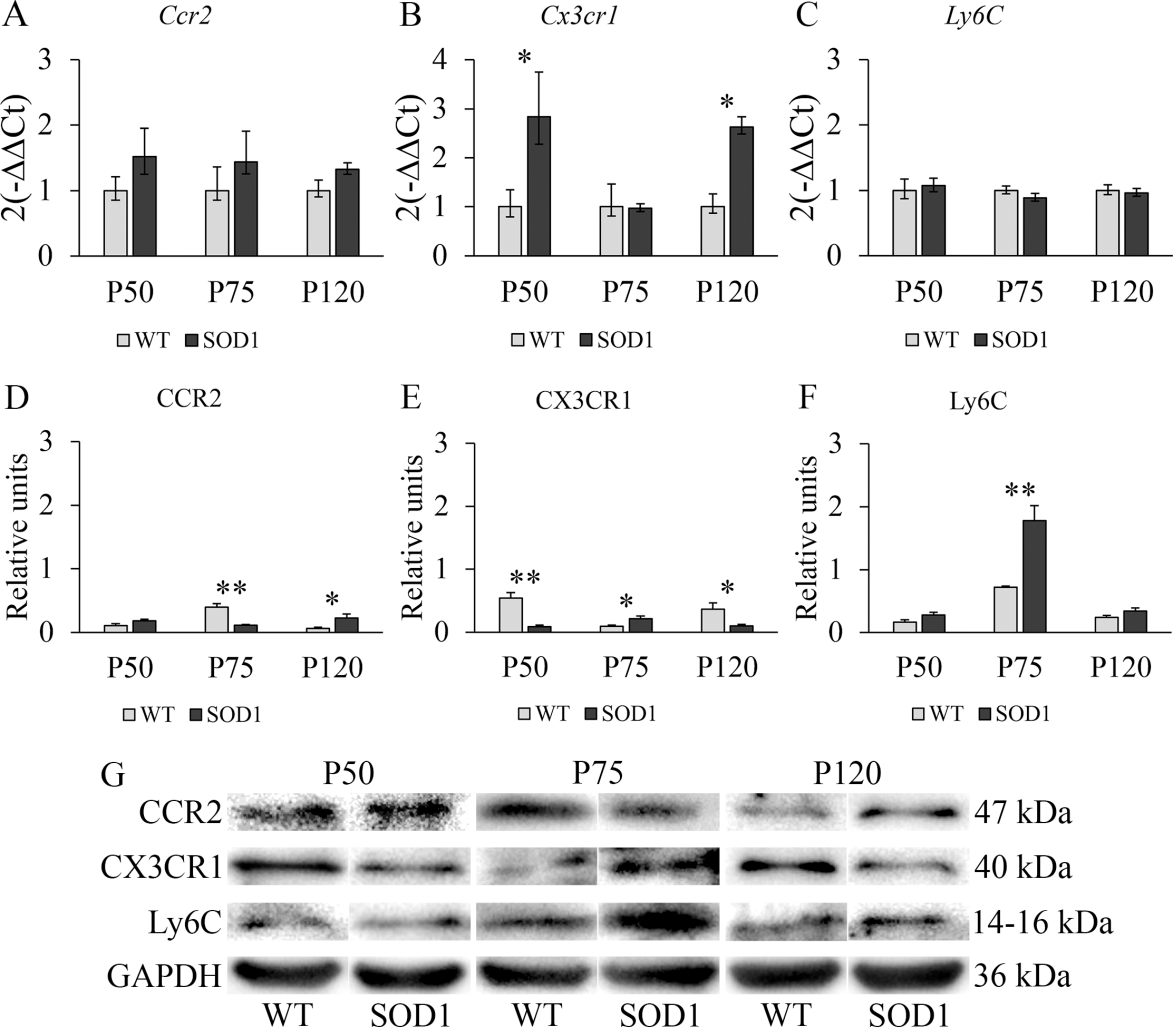
RT-PCR and Western blot study of monocytic markers CCR2, CX3CR1 and Ly6C in the skeletal muscle of WT and transgenic SOD1G93A mice. **(A, B, C)** RT-PCR was used to measure mRNA expression levels of *Ccr2*, expressed in inflammatory monocytes; *Cx3cr1*, a marker of non-inflammatory monocytes; and *Ly6C*, expressed in inflammatory monocytes. **(D, E, F)** Western blot analysis of CCR2, CX3CR1 and Ly6C protein expression levels. *y*-axis and *x*-axis represent the gene expression fold change and time points selected for RT-PCR analysis, and protein fold change and time point selected for Western Blot analysis, respectively. Transgenic SOD1G93A mice are represented with black bars and WT litter mates used as controls, with light grey bars. Error bars represent the standard error of the mean (s.e.m.) for the Western blot analysis and error bars for the RT-PCR analysis were calculated using the method developed by Livak et al. [30]. Statistical significance was established at *p*-values under 0.05 (*) and high statistical significance at *p*-values under 0.01 (**). n = 12 animals per genotype and time point. **(G)** Representative protein bands for every protein studied by Western blot in both WT and SOD1G93A mice. GAPDH was used as housekeeping protein for protein normalization.

The statistical assessment indicated a significant interaction effect between genotype and time point variables for *Cx3cr1* gene (F(2,66) = 6.328, *p* < 0.05, η^2^ = 0.395) expression levels and also on CCR2 (F(2,66) = 15.99, *p* < 0.001, η^2^ = 0.627), CX3CR1 (F(2,66) = 10.61, *p* < 0.01, η^2^ = 0.516) and Ly6C (F(2,66) = 8.5, *p* < 0.01, η^2^ = 0.459) protein levels. Fig 4 shows that mRNA levels of the three genes selected did not show any significant variation in the main stages of the disease, except for an increase in *Cx3cr1* levels at P50 and P120 in the SOD1G93A mice (*p* < 0.05) (Fig 4B). Notwithstanding, CCR2, CX3CR1 and Ly6C protein levels presented a similar scenario as the one previously observed in the liver at P75 (Fig 4D, 4E and 4F). The downregulation of CCR2 in this stage of the disease (*p* < 0.01) could suggest, as in the case of the liver, a potential differentiation of inflammatory monocytes to inflammatory macrophages. At the same time, the upregulation of CX3CR1 and Ly6C levels in the SOD1G93A mice (*p* < 0.05 for CX3Cr1 and *p* < 0.01 for Ly6C) could indicate that the muscle is receiving a higher input of non-inflammatory monocytes from blood, as well as these monocytes being able to differentiate to their respective macrophage type as they enter the damaged muscle tissue. Additionally, at P120, an upregulation of CCR2 levels, together with a downregulation of CX3CR1 levels (*p* < 0.05 for both cases) could prompt further inflammation in contrast to the balanced response to inflammation observed in the liver. Consequently, significantly lower CX3CR1 protein levels in transgenic SOD1G93A mice at P120 could prevent a regulatory response to compensate for the inflammatory process. Considering that the skeletal muscle is a non-lymphoid tissue but it is a site of active HSPCs trafficking from blood to lymph, our next step was to analyze the efficiency of the bone marrow to release HSCs, CLPs and CMPs to the blood stream to investigate if a deregulation in this process could influence the response observed in the skeletal muscle.

### HSCs were efficiently released at the terminal stage in the transgenic SOD1G93A mice

**Fig 5:**
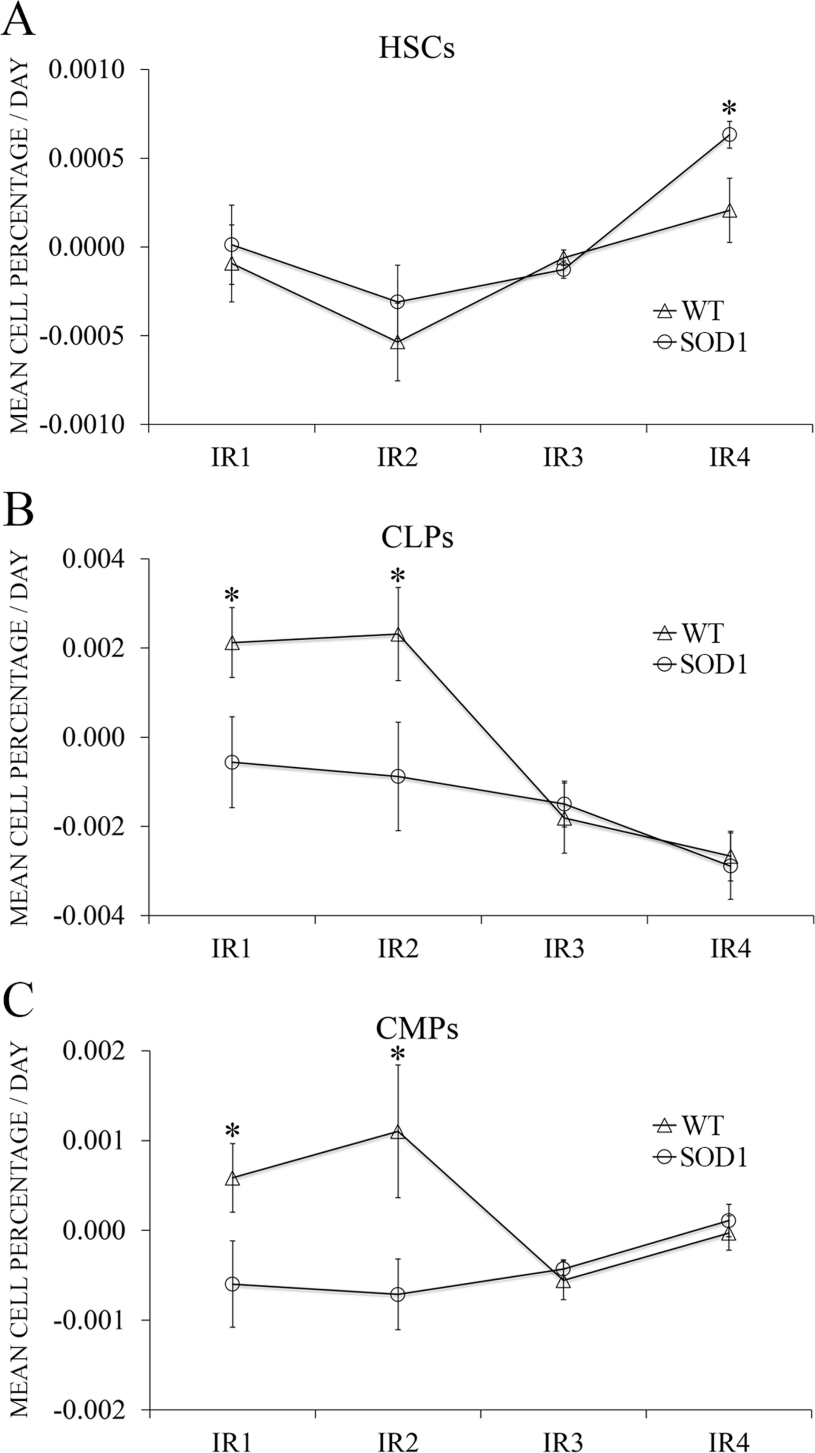
Input Rate (IR) differences between WT and SOD1G93A mice along time. Each IR represents the difference between the cell percentage at one given time point and the preceding one, divided by the days elapsed between the two blood extractions. *y*-axis represents the mean cell percentage released per day for each cell type studied, while *x*-axis represents each IR. Control mice are represented with rhombuses and SOD1G93A mice with squares. The IR was used to assess the release efficiency of **(A)** HSCs, **(B)** CLPs and **(C)** CMPs to the blood stream. IR values are represented as percentage means per day ± s.e.m. Asterisks represent an independent t-test *p*-value < 0.05 (*); n = 12 animals per genotype (extractions were performed serially along time, always using the same mice).

Input rates (IR) assessing the release of HSPCs from the bone marrow to blood were then calculated and analyzed as described in the corresponding Material and Methods subsection. Results from the two-way mixed ANOVA indicated that there was a significant difference between the four time points selected in the case of CLPs and CMPs (F(3,22) = 6.47, *p* < 0.05, η^2^ = 0.312 and F(3,22) = 6.83, *p* < 0.05, η^2^ = 0.345, respectively) and also between both genotypes for the three cell types (F(1,22) = 5.73, *p* < 0.05, η^2^ = 0.315 for HSCs, F(1,22) = 7.86, *p* < 0.05, η^2^ = 0.493 for CLPs and F(1,22) = 7.53, *p* < 0.05, η^2^ = 0.450 for CMPs). HSCs input rates were the same for both WT and SOD1G93A mice with exception of IR4 (corresponding to the terminal stage), when SOD1G93A mice were more efficiently releasing HSCs to the blood stream (*p* < 0.05) (Fig 5A). On the contrary, both CLPs and CMPs showed significantly different IR1 and IR2 (corresponding to the presymptomatic stage) (*p* < 0.05), with SOD1G93A mice releasing CLPs and CMPs slower than WT mice to the blood stream. No statistical differences were found for CLPs and CMPs for IR3 and IR4 (corresponding to the symptomatic and terminal stage) (Fig 5B and 5C). These findings could indicate that, at the terminal stage, HSCs could be more efficiently released because they exert and effect in the extravascular tissues, especially the skeletal muscle. The lower input rates found for CLPs and CMPs in the SOD1G93A mice could be related to their exit from the blood instead of a lower release from the bone marrow, as we did not observe a reduction of the myeloid population in the extravascular tissues studied. In light of these results, and taking into consideration the relevant role of monocytes in this animal model along disease progression, we analyzed the potential prognostic nature of monocytes in blood.

### Both inflammatory and non-inflammatory monocytes correlated with the survival of the transgenic SOD1G93A mice

**Fig 6:**
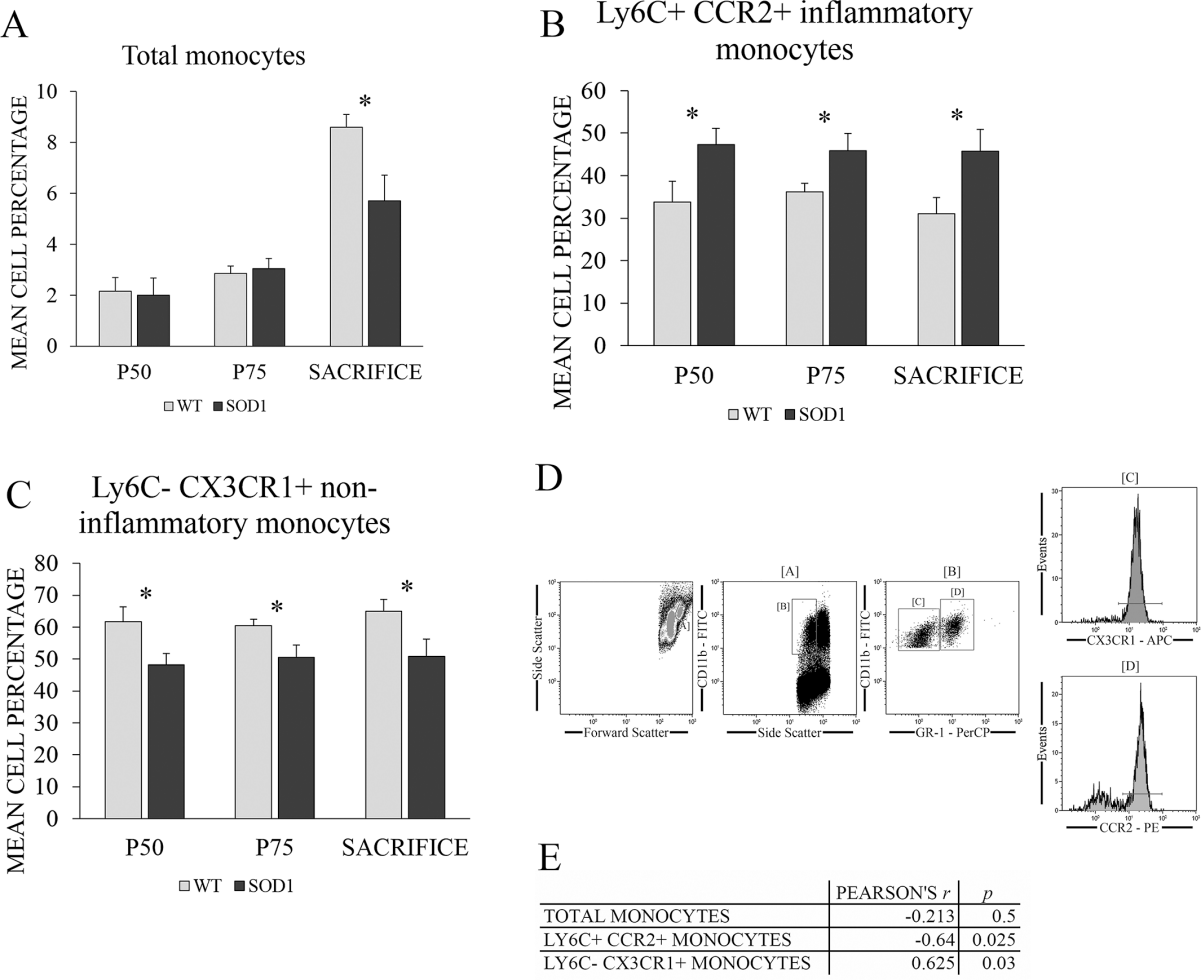
Potential role of monocytes in the survival of transgenic SOD1G93A mice. **(A)** Total monocytes, **(B)** inflammatory and **(C)** non-inflammatory monocytes percentages were measured by flow cytometry in the blood of WT (light grey bars) and transgenic SOD1G93A mice (black bars) at the main stages of the disease: asymptomatic stage (P50), symptomatic stage (P75) and terminal stage (mice endpoint ranged between 128 and 143 days, depending on the survival of the individual animals). WT litter mates were used as controls. *y*-axis represents the mean cell percentage for each cell type studied, while *x*-axis represents the time points selected for blood extraction. The gating strategy of the flow cytometry analysis is shown in **(D)**. Finally, the correlations found between monocyte types and survival is shown in **(E)**. Data are presented as means and error bars represent the standard error of the mean (s.e.m.). Statistical significance was established at *p*-values under 0.05 (*). n = 12 animals per genotype (extractions were performed serially along time, always using the same mice).

To finally investigate the potential role of both monocyte populations in the survival of transgenic SOD1G93A mice, inflammatory and non-inflammatory monocyte populations were measured by flow cytometry at the main stages of the disease (Fig 6), following the gating strategy shown in Fig 6D. Results from the two-way mixed ANOVA indicated that there was a significant difference between the three time points selected (F(2,22) = 38.52, *p* < 0.001, η^2^ = 0.637) and also between both genotypes (F(1,22) = 5.59, *p* < 0.05, η^2^ = 0.320) when referring to total monocyte population. Also, an interaction between both variables existed (F(2,22) = 3.72, *p* < 0.05, η^2^ = 0.144). Concretely, total monocyte population was significantly decreased at sacrifice in transgenic SOD1G93A mice (*p* < 0.05) (Fig 6A). In the case of inflammatory and non-inflammatory monocytes, no overall differences were found between time points. Also, no significant interaction was found between both varibles. However, and most importantly, a overall significant difference existed in the case of both genotypes (F(1,22) = 9.68, *p* < 0.01, η^2^ = 0.306 for inflammatory monocytes and (F(1,22) = 9.81, *p* < 0.01, η^2^ = 0.309) for non-inflammatory monocytes). More specifically, a steady increase of inflammatory monocytes together with a steady decrease of non-inflammatory monocytes along disease progression in the SOD1G93A mice (*p* < 0.05 for all cases) suggested the presence of and ongoing inflammatory process in this animal model starting at a very early asymptomatic stage (Fig 6B and 6C).

Additionally, Pearson’s *r* coefficients were calculated to study the correlation of the evolution (slopes generated by fitting a linear regression model) of monocytes along disease progression with the survival of the animals (Fig 6E). For this purpose, first, the individual slopes for each mouse were calculated from the monocyte percentages at the three time points selected (P50, P75 and sacrifice). These slopes were then correlated with the individual survival values of each mice in the overall Pearson’s correlation calculation. No significant correlation with the survival of SOD1G93A mice was observed in the case of total monocytes. However, the slopes of inflammatory and non-inflammatory monocytes correlated significantly with survival of SOD1G93A mice. A strong negative correlation was found between variation of inflammatory monocytes along disease progression and survival of mice (*r* = -0.640, *p* < 0.05), while a positive correlation was found in the case of non-inflammatory monocytes (*r* = 0.625, *p* < 0.05). This means that the higher the percentage of inflammatory monocytes, the shorter the survival of SOD1G93A mice, and the same applies the other way around. Also, both squared Pearson correlation coefficients were around 0.4, which implies that 40% of the variation in one variable could be explained by their association with the other. In the case of the non-inflammatory population of monocytes, a higher percentage of this population meant a longer mice survival, and vice versa. This could be in accordance with the hypothesis that a higher population of non-inflammatory monocytes and their macrophage descendants could compensate for further inflammation in the tissues, as it was previously observed in the liver. However, more studies are needed to correctly establish the direction of the relationship (i.e., the causality) between both variables, as correlation coefficients do not have the power to indicate it. Nonetheless, what is clear is that longer-lived mice had less inflammatory and higher non-inflammatory monocytes than the shorter-lived mice, so that the results found revealed that inflammatory and non-inflammatory monocytes could be used as potential biomarkers of longevity in blood of transgenic SOD1G93A mice.

## Discussion

Understanding the potential role of the hematopoietic and immune system in the progression of a disease of unclear ethiopathology such as ALS could enable the identification of novel biomarkers and, therefore, more conducted and specific therapeutic strategies based on HSPCs and monocytes could be designed. In particular, ALS has lately become a suitable scenario to study the interplay between the hematopoietic and immune system and disease progression. Recent studies in *C9orf72* null mice have demonstrated that the loss of *C9orf72* prompted alterations in macrophages and microglia together with an age-related neuroinflammation similar to *C9orf72* ALS [33]. Our main aim in this study was to analyze the role of HSPCs and monocytes in the disease progression of the most commonly used ALS murine model, the transgenic SOD1G93A mice.

For this purpose, we first studied HSPC populations in the spleen of transgenic SOD1G93A mice. The spleen is considered a secondary lymphoid organ which could potentially contribute to the monocyte role in ALS. It is a known reservoir of monocytes and it also presents the ability to direct their differentiation [14, 34]. In this sense, HSC, CLP and CMP populations were analyzed by flow cytometry in the spleen of transgenic SOD1G93A mice at the asymptomatic (P50), symptomatic (P75) and terminal stages (P120) of the disease. The results showed an alternative lineage differentiation of HSCs in transgenic SOD1G93A mice at every stage of the disease. The lymphoid lineage was favoured at P75, while myeloid lineage was increased at the terminal stage, together with a significant upregulation of HSC population. This finding suggested that a preferential myeloid differentiation of HSCs in the spleen could be promoted by the advanced state of the disease progression. The complete absence of CLPs in SOD1G93A at P120 could explain the lymphopenia observed in spleen by other authors at the terminal stage in the murine model [35, 36], as CLPs are the progenitor source of mature lymphocytes.

To further examine if this myeloid differentiation could be translated into an inflammatory response based on inflammatory monocytes, we analyzed the gene and protein levels of CCR2, CX3CR1 and Ly6C in the spleen at the same stages of the disease. Notwithstanding, the population of inflammatory monocytes was not altered in this tissue since CCR2 protein levels remained overall unchanged. Additionally, CX3CR1 and Ly6C protein levels were found to be significantly downregulated only at P120. Consequently, these findings could suggest that inflammatory monocytes are not infiltrating the spleen, and some of them may even be leaving it at P120, as evidenced by the light decrease in Ly6C protein levels. On the other hand, non-inflammatory monocytes seem to be leaving the spleen at P120.

A non-lymphoid organ that contributes to the inflammatory and non-inflammatory monocyte role in ALS is the liver. This organ is the main producer of acute phase and complement proteins as well as cytokines and chemokines, all of them related to inflammation and leukocyte chemotaxis. It also possesses a critical immunosurveillance role, being the tissue with the greatest population of specific resident macrophages, known as Kupffer cells, and also Natural Killer cells (NK cells), Natural Killer T cells (NKT cells) and reticuloendothelial cells [36, 37]. Interestingly, at P75, the only significant alterations were observed in *Ccr2* and *Cx3cr1* mRNA levels, which decreased significantly, while the protein profile levels showed the contraty scenario in transgenic SOD1G93A mice. The significant upregulation of CCR2 and CX3CR1 protein levels at P50 suggests the presence of both inflammatory and non-inflammatory monocytes at a very early stage of the disease. However, the downregulated CCR2 and CX3CR1 protein levels at P75 could be due to their exit to the blood or to a potential differentiation to inflammatory macrophages, as the surface expression of CCR2 diminishes as inflammatory monocytes differentiate to macrophages [32]. In connection with these results, it has been demonstrated that the liver shows pathological changes in the murine model, including a progressive parenchyma atrophy, mass loss, alterations of the IGF-1 axis and increase of NKT cells [36]. In addition, it is know that this organ responds with a high recruitment of circulating immune cells when inflamed [38]. At P120, the downregulated CCR2 protein levels and the upregulated CX3CR1 and Ly6C protein levels could indicate the entering of non-inflammatory monocytes and their possible differentiation to non-inflammatory macrophages *in situ*, as described in other works [39]. The slight increase of Ly6C could be due to the presence of T cells instead of inflammatory monocytes, as CCR2 is downregulated) and 50% T cells express the surface receptor Ly6C [40]. Non-inflammatory monocytes and macrophages in the liver can enter and be originated, respectively, after liver injury, which is in accordance with previous studies [31]. Consequently, the liver of transgenic SOD1G93A mice seemed to operate in a regulatory loop along disease progression and, in spite of the neurodegenerative processes of the disease, it could maintain homeostasis relatively well.

In the light of this different behaviour of monocytes in the spleen and liver, we wondered if the skeletal muscle, which is one of the most affected tissues by the disease progression, could be an active monocyte trafficking site. In contrast to the liver, at P75 the inflammatory monocytes seemed to be decreased in the skeletal muscle or hypothetically differentiating to inflammatory macrophages, as shown by the decreased CCR2 protein levels in the SOD1G93A mice. On the other hand, the increase of CX3CR1 indicated the recruitment of non-inflammatory monocytes to the skeletal muscle. As observed in the liver, the presence of increased Ly6C levels but decreased CCR2 protein levels could indicate the infiltration of T lymphocytes to the skeletal muscle, as they also possess a prominent role in the inflammatory process observed in ALS [40]. Even though non-inflammatory monocytes were seemingly being recruited at P75, lower CX3CR1 protein levels were found in transgenic SOD1G93A mice at P120, and this could prevent a regulatory response to compensate for the inflammatory response, which is more prominent at the symptomatic and terminal stages of the disease. These findings were in accordance with the fact that a lack of differentiation towards the non-inflammatory subset could promote inflammatory monocyte proliferation, which could be explained by the damaged tissues specifically recruiting inflammatory monocytes [15, 41].

With respect to the mismatch we observed between mRNA and protein levels, especially in the liver and the skeletal muscle, this phenomenon has been widely reported in many studies. Lately, it has been accepted that the ratios of protein levels do not show a one-to-one correlation with the ratios of mRNA levels. This is because the relation between transcription and translation is very complex, and not straightforward [42–44]. The lack of correlation between mRNA and protein levels is believed to be due to several potential causes. First, there are many complex post-transcriptional mechanisms involved in the step between mRNA and protein which are not well-known or defined. Also, proteins may have very different half-lives, so that when measured, the concentration obtained does not reflect the original one. Finally, proteins also suffer many different post-translational modifications which could affect their expected levels [43, 45]. All potential causes could be involved in the differences we found between the mRNA levels and the protein levels in the liver and the skeletal muscle, as these monocytes can be very dynamic and can change the expression of the different surface proteins characterizing their population type, for example, with inflammatory monocytes losing expression of Ly6C and CCR2 and highly expressing CX3CR1, thus giving rise to non-inflammatory subsets.

In relation with HSPCs, and considering the fact that tissue HSPCs can be replenished by the pool of blood HSPCs [46], our next step was to analyze the efficiency of the bone marrow to release HSCs, CLPs and CMPs to the blood stream to investigate if a deregulation in this process could influence the cell response observed, especially in the skeletal muscle. Input rate (IR) was defined as a new parameter to measure HSPCs release efficiency. At the terminal stage, the transgenic SOD1G93A mice released HSCs more efficiently (more cells per day) than the WT mice, as proved by the increased IR4 value, suggesting that HSCs could be entering the blood in bigger numbers to assist the damaged tissues or to differentiate and give rise to a high number of immune system cells, including monocytes [39]. However, in the presymptomatic stage, CLPs and CMPs were released less efficiently in transgenic SOD1G93A mice than in the WT mice. Taking into account the previous results in spleen, liver and skeletal muscle, the IR value decrease for CLPs and CMPs in the asymptomatic stages of the disease could be due to their recruitment into the extravascular tissues affected by the disease, and not to a deficient release from the bone marrow to the blood.

The different interplay among the hematopoietic and immune system and the spleen, the liver and the skeletal muscle along disease progression in the transgenic SOD1G93A mice enhanced our study to unravel the potential prognostic nature of monocytes in blood. The immune system has been repeatedly demonstrated to be activated in ALS, including changes in monocyte receptors and monocyte activation, as well as affecting monocyte descendants, the macrophages [18, 20, 47]. The changes observed in peripheral blood could be related to the immune system following the call of injured tissues, thus releasing more monocyte precursors (HSCs and CMPs), as well as more inflammatory monocytes from the bone marrow in a CCR2-dependent manner [48], and increasing blood levels of inflammatory monocytes in detriment of non-inflammatory monocytes. In this sense, inflammatory and non-inflammatory monocyte populations in blood from transgenic SOD1G93A mice were measured by flow cytometry at the main stages of the disease. The results showed that inflammatory monocytes were significantly increased along disease progression in the SOD1G93A mice compared to the controls, starting at P50. Notwithstanding, non-inflammatory monocytes remained decreased in the SOD1G93A mice compared to the WT mice along all the disease progression. Furthermore, a negative correlation was found between inflammatory monocytes and the longevity of SOD1G93A mice during disease progression, so that the higher the percentage of this population of monocytes, the shorter the survival of the animals, and viceversa. Although the immune system tries to resolve the pathology observed in the disease, the exacerbation of the inflammatory state is clearly not aiding the survival but rather worsening it. Some works demonstrated that a decrease in CCR2, which further identifies and purifies Ly6^hi^ inflammatory monocytes, predicted a better outcome in ALS patients, as the inflammatory monocyte activation seemed to be more destructive than protective and accelerated the disease progression, thus resulting in a worse prognosis [49]. The same detrimental effect has been observed in other pathologies such as multiple sclerosis [50], ischemic brain injury [51], atherosclerosis [52] and arthritis [23], among others. Interestingly, a positive correlation was found between non-inflammatory monocytes and survival of SOD1G93A mice, with a higher percentage of this monocyte population resulting in a longer survival of the animals, and vice versa. An increment of circulating non-inflammatory monocytes could also favour an increment of these cells in trafficking sites in physiological and pathological conditions, such as the liver or the skeletal muscle. On the other hand, the predominance of non-inflammatory monocytes could potentially lead to a better outcome, due to the capacity of this population to enter the damaged tissues in a CX3CR1-mediated manner to repair tissue damage, secrete anti-inflammatory cytokines, and promote regeneration [14, 16]. This positive effect has been demonstrated in myocardial infarction, where non-inflammatory patrolling monocytes enter the cardiac muscle to recover damage and promote angiogenesis [53, 54]. Non-inflammatory monocytes also act in neurodegenerative diseases like Alzheimer’s disease, where they remove amyloid-β peptides from the brain vasculature [55], and are also known to prevent excitotoxicity [56], which could also explain the positive correlation found with survival, as glutamate-induced excitotoxicity is one of the pathological mechanisms found to participate in ALS pathogeny. Finally, they also play an important role in muscle regeneration, as a correct CCR2+ inflammatory to CX3CR1+ anti-inflammatory monocyte differentiation ratio is critical for muscle regeneration following injury, with CX3CR1 anti-inflammatory monocytes being the ultimate repairing monocytes [57, 58], so that their increased percentage could promote a longer survival in the SOD1G93A mice model by contributing to muscle regeneration. However, care must be taken when interpreting these results, as correlation coefficients do not have the power to indicate the direction of the relationship (i.e., the causality) between the variables implicated, so while an association between them can be established, exactly how both variables affect each other remains to be elucidated in future studies. Nonetheless, what does seem clear is that longer-lived mice had less inflammatory and more non-inflammatory monocytes than the shorter-lived mice, so that the results found revealed that inflammatory and non-inflammatory monocytes could be used as potential biomarkers of survival in blood of transgenic SOD1G93A mice.

In summary, our findings suggest a close interplay between HSPCs and monocytes and disease progression in transgenic SOD1G93A mice. The inflammatory response due to the neurodegeneration in this animal model affected secondary lymphoid and non-lymphoid organs and tissues, especially the liver and the skeletal muscle. However, the non-inflammatory monocytes could be responsible of the resolution state observed in the liver of this animal model. In contrast, in the skeletal muscle, inflammatory monocytes prompted a further inflammation state up to the terminal stage (P120) of the disease. Finally, in blood, a positive correlation was found between percentage of non-inflammatory monocytes and survival of SOD1G93A mice, while the contrary association (negative correlataion) was found for inflammatory monocytes. Therefore, both monocyte populations could be considered prognostic biomarkers of survival in this animal model. These findings could pave the way to translational studies in ALS patients, promoting the identification of new reliable biomarkers of disease progression at the clinical level and, consequently, new promising therapeutic strategies.

## Supporting information

S1 Dataset This supporting information file contains relevant monocyte raw data used to reach the described conclusions. (XLSX)
